# Marker-independent vibrational spectroscopy imaging recognizes the hypoxia effect in the human brain endothelium

**DOI:** 10.1038/s41598-025-11000-2

**Published:** 2025-07-18

**Authors:** Aleksandra Pragnąca, Anna Antolak, Zuzanna J. Krysiak, Monika Leśniak, Agata Borkowska, Robert Zdanowski, Kamilla Malek

**Affiliations:** 1 Department of Chemical Physics, Faculty of Chemistry, Jagiellonian University in Krakow, Gronostajowa 2, 30-387 Kraków, Poland; 2 Doctoral School of Exact and Natural Sciences, Jagiellonian University in Krakow, prof. S. Lojasiewicza 11 Street, 30-348 Kraków, Poland; 3 Laboratory of Molecular Oncology and Innovative Therapies, Military Institute of Medicine National Research Institute, Szaserow 128 Street, 04-141 Warsaw, Poland; 4 Department of Biosystems and Soft Matter, Institute of Fundamental Technological Research, Polish Academy of Sciences, Pawińskiego 5B, 02-106 Warsaw, Poland

**Keywords:** Hypoxia, Brain endothelium, FTIR and Raman spectroscopy imaging, Spectral markers, Biophysical chemistry, Infrared spectroscopy, Raman spectroscopy, Imaging and sensing

## Abstract

Brain microvascular endothelial cells experience hypoxic conditions in several neurodegenerative disease processes and the underlying mechanisms still need to be explored. Current imaging modalities and biochemical assays require many specific markers that should be detected to identify the hypoxic response, especially at a level of single cells. This study presents a single-cell molecular imaging approach utilizing Fourier-Transform Infrared and Raman spectroscopy. Those methods enable the simultaneous detection of proteins, lipids, and nucleic acids encoded in their unique vibrational fingerprints. By establishing ratiometric estimators, we measured upregulated lipid metabolism, structural changes of proteins and asses DNA:RNA ratio at the single-cell level induced by oxygen depletion. Moreover, this approach allows for analyzing changes within specific cellular compartments, including nuclei, providing a comprehensive understanding of how hypoxia affects cellular functions and metabolism. Our findings pave the way for future investigations into the cellular adaptations to hypoxia in brain endothelial cells, potentially revealing novel therapeutic targets for neurodegenerative diseases.

## Introduction

Hypoxia is a condition in which the oxygen content in the blood is reduced, or the process of oxygen O_2_ delivery to cells is impaired, resulting in a decrease in the partial pressure of oxygen at the tissue level^1^. It is intrinsically linked to neurological disorders, including Alzheimer’s, Parkinson’s, and other age-related neurodegenerative diseases^2–4^. Hypoxia and oxygenation, which occur in various pathological conditions, affect the blood–brain barrier (BBB)—a selective semi-permeable membrane between the blood and the central nervous system (CNS).

Brain endothelial cells are significantly more sensitive to hypoxia than other components of the BBB^5^. Molecular adaptations to hypoxia involve numerous signaling pathways, evoke energy metabolism response, and launch biochemical adjustments at epigenetic and metabolomic levels^6^. During hypoxia, key processes involve modulation of oxidative stress, mitochondrial dysfunction, and inflammation, with the hypoxia-inducible factor 1 (HIF-1) pathway playing a crucial role^3,7,8^. Adaptive responses to hypoxia at the cellular level are initiated by signaling molecules, including reactive O_2_ species (ROS) and transcription factors^9^. In the case of brain endothelium, hypoxia induces modification of its phenotype, and in consequence, it leads to capillary leakage, prothrombotic activation, and initiation of the inflammatory reaction^10^. HIF-1 causes activation of the vascular endothelial growth factor (VEGF) in endothelial cells, which is responsible for vascular leakage in the brain^11^, which subsequently affects vascular permeability by fragmentation of the endothelium, degenerative changes in the endothelial basement membrane, and changes in tight junction protein expression^12,13^.

Hypoxia affects cells by altering gene expression, protein modifications, and cellular processes^6^. It leads to the accumulation of unfolded proteins in the endoplasmatic reticulum (ER)^14^, oxidative modifications of cysteine residues^15^, and changes in protein structure and function due to ROS^16^. Hypoxia also inhibits protein translation but allows for the synthesis of proteins critical for hypoxic adaptation^17^. In addition, it promotes perinuclear mitochondrial localization and induces mitochondrial fission, contributing to the cell’s response to low oxygen conditions^18,19^. These changes disrupt cellular homeostasis, impacting membrane integrity and overall function. HIF-1 ultimately promotes fatty acid synthase expression, which damages cellular structures. To address this issue, cells adjust their triglyceride composition by accumulating surplus saturated fatty acids in lipid droplets and releasing unsaturated oleate to maintain the balance of membrane phospholipid saturation^20^.

The hypoxic conditions are used to model neurodegenerative diseases in vitro. Fundamental knowledge about the subcellular processes that occur inside a single cell under disease conditions is crucial for understanding the mechanism of pathology development and potential therapeutic applications for neurodegenerative diseases. Although the bases of hypoxic effects have been outlined in some detail, emerging research areas focus on exploring how oxygen availability impacts multiple distinct but interdependent adaptations at the cellular level.

Detecting hypoxia in the brain cells is essential for understanding cellular adaptations to low oxygen levels, but currently used methods have limitations. Bioassays like Western Blotting and ELISA detect proteins associated with hypoxia, such as HIF-1α, VEGF, and GLUT-1^21^. However, they only offer indirect evidence, often require large sample sizes, and are unsuitable for single-cell analysis. Immunofluorescence microscopy and flow cytometry localize markers of hypoxia at the single-cell level and in large populations, respectively; however, several obstacles, e.g., conditions of cells, fixation, permeabilization, antibody specificity, multiplex staining, and real-time monitoring, make them difficult, time-consuming, and expensive. Targeting pre-selected proteins relies on the availability and accuracy of antibodies, which cannot reveal unexpected or broad changes, or simply that hypoxia can induce protein modifications and their isoforms that are unrecognized by antibodies. Quantitative analysis is also complex due to detection sensitivity, autofluorescence, and cellular morphology alternation. Quantitative Real-Time PCR assesses mRNA levels of hypoxia-related genes (HIF isoforms, VEGF)^22^ and identifies the transcriptional response. However, it does not directly measure the protein levels, which may not correlate with mRNA expression due to post-transcriptional modifications. Moreover, hypoxia can affect the expression of housekeeping genes commonly used for normalization, which introduces additional variability. Non-coding RNAs and RNA modifications, which play essential regulatory roles under hypoxic conditions, are often overlooked in standard qPCR. Assays incorporated with a Seahorse XF Analyzer detect changes in glycolysis and oxidative phosphorylation; but, this instrument is costly, and data interpretation remains a big challenge. A detailed molecular profile of dysfunctional cells by quantifying metabolites (e.g., Krebs cycle intermediates)^23^ and proteins can be determined by sensitive and specific mass spectrometry after destructive sample preparation, including cell lysis, protein digestion, and purification. Despite a broad methodology being offered to follow hypoxia induction, these approaches are time- and cost-consuming and can be obscured by undesired effects induced by this stress condition.

This variety of crucial markers and bioassays shows how complex the examination of the hypoxic effect is, and new analytical methods offering non-destructive sample preparation without tags are still sought. In this work, we propose including vibrational spectroscopy microscopy to detect the hypoxic impact on the cerebral microvascular endothelial cells. Fourier transform Infrared (FTIR) and Raman (RS) spectroscopies offer the in situ detection of major cellular biomolecules like proteins, lipids, and nucleic acids by directly probing the cell body and its compartments. We determined spectral differences between brain endothelial cells (HBEC 5i cell line) in normoxic and hypoxic conditions. We quantified them and provided a marker set, which can be associated with the already-identified biochemical effects of the oxygen depletion. The high spatial resolving powers of IR and Raman microscopes revealed substantial chemical changes in the nucleus and surrounding compartments. Still, we also showed that they are observable using low-resolution IR microscopy. This work demonstrated the versatility of choosing accessible IR and Raman tools to monitor the markers established by us in the cells subjected to hypoxic stress. The simplicity of data collection and the proposed methods of analysis can be particularly useful as the first screening strategy in hypoxia-related studies, indicating which cellular compartments and biomolecules are involved in this stress, and thus guiding researchers toward targeted analyses, providing a deeper insight into molecular biology.

## Materials and methods

### Cell culture

Cerebral microvascular endothelial cells (HBEC 5i) were purchased from ATCC (CRL-3245, USA) and cultured up to the 9th passage in DMEM/F12 (Gibco, UK) medium supplemented with 10% fetal bovine serum (FBS, Gibco, UK), 1 × antibiotics (100 × Penicillin–Streptomycin, Gibco, UK) and 400  µL of endothelial cell growth supplement (ECGS 5 mg/mL, Sigma-Aldrich, USA) on tissue culture plates coated with 0.1% of gelatin solution (Sigma-Aldrich, USA). The cell culture medium was changed twice a week.

### Normoxia

CaF_2_ Raman grade glass slides (Ø 12 mm by 1 mm, Crystran, UK) were sterilized, placed in the 24-well plate and coated with 0.5 mL of poly-L-lysine (1mg/mL, Sigma-Aldrich, USA) solution for at least 1 h in 37 °C, then it was discarded. Glass slides were washed twice with sterile dH_2_O and then twice with Dulbecco’s Phosphate Buffered Saline (PBS, Corning, USA), to remove the traces of coating solution. HBEC 5i cells were placed in the incubator and cultured at 37 °C and humidity of 90% in a 5% CO_2_ atmosphere for 24 h (NuAire incubator, USA). Next, 1.2 × 10^5^ cells per sample were seeded, cultured for 5 h, and then washed 3 times with PBS to remove the medium. 0.5 mL of 2.5% glutaraldehyde (Chempur, Poland) solution was added and incubated for 5 min at RT to fix the cells. Then, the fixing solution was removed, and samples were washed 3 times with PBS and stored at 4 °C.

### Hypoxia

HBEC 5i cells were placed in the XVivo X3 workstation (Biospherix, Parish, NY, USA) with a 1% O_2_ concentration 24 h before seeding on CaF_2_ Raman-grade glass slides. This station provides precise control over oxygen levels, ensuring a stable and reproducible hypoxic environment. Notably, the utilization of this equipment eliminates the need for examining additional hypoxia markers, streamlining experimental processes. The cell culture medium was changed after being stored under hypoxic conditions for 24 h. After 24 h in hypoxia, cells were seeded on CaF_2_ Raman-grade glass slides and processed according to the protocol previously described for normoxia. The whole procedure was performed under 1% O_2_ concentration.

### Immunostaining

Glass slides (Ø 12 mm, Bionovo, Poland) were sterilized, placed in the 24-well plate, and coated with 0.5 mL of 0.1% gelatin solution for at least 45 min at 37 °C, after that time gelatin was removed. HBEC 5i were cultured on the glass slides at 37 °C and humidity of 90% in a 5% CO_2_ atmosphere (NuAire incubator, USA) for 24 h, with the cell density 9 × 10^5^ per sample. Next, the cell culture medium was removed and cells were fixed with 1.5% glutaraldehyde for 1 h at 4 °C. The samples were washed 3 times with PBS and permeabilized with 1 × Blocker BSA in TBS (Thermo Fisher Scientific, UK) for 1 h at 4 °C. The solution was removed and cells were again washed 3 times with PBS and incubated with 200 µL of BODIPY (Thermo Fisher Scientific, USA) (10 µm) for 15 min at RT. Then, samples were washed 3 times with PBS and counterstained with Alexa Fluor™ 647 Phalloidin (100 × diluted with PBS) for 1h at RT. The dye was removed, samples were washed with PBS and stained with Hoechst (1000 × diluted with PBS, Thermo Fisher Scientific, UK). Finally, they were mounted to the microscope slide using Dako Fluorescence Mounting Medium (Agilent, USA). Zeiss Axio Observer with Axiocam 503 mono and Apotome 3 (Carl Zeiss Microscopy GmbH, Germany) was used to image fixed samples. Images were acquired using ZEN microscopy software (Carl Zeiss Microscopy GmbH, Germany). 395 nm, 495 nm and 660 nm laser lines were used for excitation, and emission detection bands were 420–470 nm for Hoechst and 500–550 nm for BODIPY and 665–715 for Phalloidin.

### Cell cycle analysis

Tali® Cell Cycle Kit (A10798, Molecular Probes by Life Technologies) was used to evaluate the cell cycle of endothelial cells. Cells were prepared for labeling according to manufacturer instructions to quantify the cellular DNA. In brief, cells were washed by DPBS (once, 2 mL) and centrifuged (300×*g*, 5 min). Then, cells were fixed with ice-cold 70% ethanol and stored at − 20 °C for at least 24 h. Before staining, cells were centrifuged (1000×*g*, 5 min, 4 °C), resuspended in 1 mL of DPBS, centrifuged (500×*g*, 5 min, 4 °C), and resuspended in 200 µL of Tali® Cell Cycle Solution (containing propidium iodide, RNase A, and Triton® X-100). Cells were incubated at RT for 30 min in the dark. Then, the cells were gently mixed on a vortex before cell cycle analysis using a cytometer (Beckman Coulter CytoFLEX flow cytometer). At least 50,000 events per sample were collected during the measurement. Firstly, cell events considered as cell debris were excluded from the analysis using the FSC/SSC parameter. Cell populations in different cell cycle phases were determined based on PI fluorescence intensity. Cell populations were determined in individual cycle phases based on the histograms. Data analysis was performed with CytExpert software to obtain accurate estimates of the percentage of cells in each phase of the cell. Data were presented as the average for the experimental group’s ± SEM (SEM, standard error of mean) and analyzed using a two-tailed Student t-test for the variance.

### IR and Raman spectroscopy imaging

Molecular imaging was performed within 72 h after cell fixation using the WITec Alpha 300 confocal Raman microscope (WITec, Germany) and Agilent 670-IR spectrometer combined with the 620-IR microscope (Agilent, USA). Raman spectra were recorded using an excitation laser at 532 nm (power of 40 mW), an integration time of 0.5 s, and a spectral resolution of 3 cm^−1^. Laser light was focused through a 40 × water immersion objective (W Plan-Apochromat VISIR, NA = 1, Zeiss, Germany). 120 Raman images per experimental group were acquired with a step size of 1 μm. The IR microscope was coupled with a focal plane array (FPA) detector cooled with liquid nitrogen. The detector consisted of a matrix of 16,384 pixels, arranged in a 128 × 128 grid format, providing a field of view of ca. 700 μm × 700 μm (SD—Standard Definition, lens with NA = 0.62 and a projected pixel size of 5.5 µm × 5.5 µm, resulting in a total magnification of 7.3×). Six images were acquired for each experimental group (ca. 20 cells per image). Ultra-High Definition IR imaging (UHD, lens with NA = 0.81 and a projected pixel size of 0.66 µm × 0.66 µm, giving a total magnification of 60×) collected IR images of nuclei (ca. 30 nuclei per experimental group). The UHD mode in the Agilent IR microscope provides the maximum lateral resolution achievable in FPA-based IR microscopy, i.e., 7.5 and 1.9 µm at 1000 and 4000 cm^−1^, respectively. 128 and 256 scans, for SD and UHD, respectively, were co-added to collect transmission FT-IR spectra in the region of 900–3700 cm^−1^ and with a spectral resolution of 4 cm^−1^. Before IR imaging, samples were washed three times with distilled water and dried overnight in a desiccator.

### Preprocessing and data analysis

Pre-processing and chemometric analyses of spectral data sets were performed using WITec Project (WITec 5.0 software, Germany), CytoSpec (ver. 2.00.01), MatLab (R2020a, MathWorks, Natick, MA, USA), PLS_Toolbox (ver. 9.2.1, Eigenvector Research, Manson, WA, USA), OPUS (ver. 7.2.139.1294, Bruker, Billerica, MA, USA) and Origin 9.1 (ver. 2020b, OriginLab Corporation, Northampton, MA, USA) software. Raman spectra were preprocessed using a cosmic ray removal filter with a size of 3 and a dynamic factor of 8. For baseline correction, the 3rd-grade polynomial was used. Next, chemical maps were constructed based on integral intensity in spectral regions specific for organic matter (2800–3030 cm^−1^), lipids (2830–2900 cm^−1^), unsaturated lipids (3000–3030 cm^−1^), nucleic acids (780–800 cm^−1^) and cytochromes (740–760 cm^−1^). Afterward, k-means cluster analysis (KMCA) with a Manhattan distance method and randomized k-means distribution was performed to segregate the Raman image of a single cell into classes attributed to the cytoplasm, nucleus, perinuclear area (an area consisting of the endoplasmic reticulum and the mitochondria), and lipid droplets. Raman spectra extracted for KMCA classes were next truncated in the spectral region of 500–3050 cm^−1^, baseline-corrected (10 iterations), smoothed according to a Savitzky–Golay protocol (17 points), and normalized (unit vector normalization). Pre-processing of FTIR images included PCA-based denoising (15 PCs) and smoothing spectra with a Savitzky-Golay algorithm (15 points). Next, unsupervised hierarchical cluster analysis (UHCA) in 920–1770 and 2800–3070 cm^−1^ regions was performed. Spectral distance was computed as D-values, and individual clusters were extracted according to Ward’s algorithm. Based on the distribution maps of proteins (1620–1680 cm^−1^), spectra from a single cell were extracted and averaged. FTIR spectra extracted from UHCA were baseline-corrected (10 iterations), transformed into a second derivative (Savitzky–Golay algorithm, 2nd order polynomial, 13 points of smoothing), and normalized (unit vector normalization) in the whole spectra range.

Pre-processing data sets were used for unsupervised Principal Component Analysis (PCA) performed in the bioregions (500–1800 cm^−1^ and 2800–3050 cm^−1^ for Raman spectra, 720–1770 cm^−1^ and 2800–3070 cm^−1^ for second derivative FTIR spectra) using an SVD algorithm of cross-validation and 7 principal components. Scores and loadings plots were generated to show grouping and variance within Raman and FT-IR spectra of cells cultured under normoxic and hypoxic conditions.

Mean FTIR and Raman spectra extracted from the cluster maps were further used to determine spectral markers and their variations based on the bands’ integral intensities (MatLab, Unscrambler, Origin software). Statistical analysis was performed with OriginPro 2023b licensed for Jagiellonian University. First, a Shapiro–Wilk and Levene’s tests were employed to assess the normality of data distribution and homoscedasticity, respectively. For data that fulfilled these conditions, a one-way ANOVA analysis was performed. In other cases, a non-parametric Kruskal–Wallis ANOVA analysis was employed. We assessed the statistical significance of spectral differences at *p*-value thresholds of *p* < 0.05, *p* < 0.01, and *p* < 0.001.

## Results and discussion

**Fig. 1:**
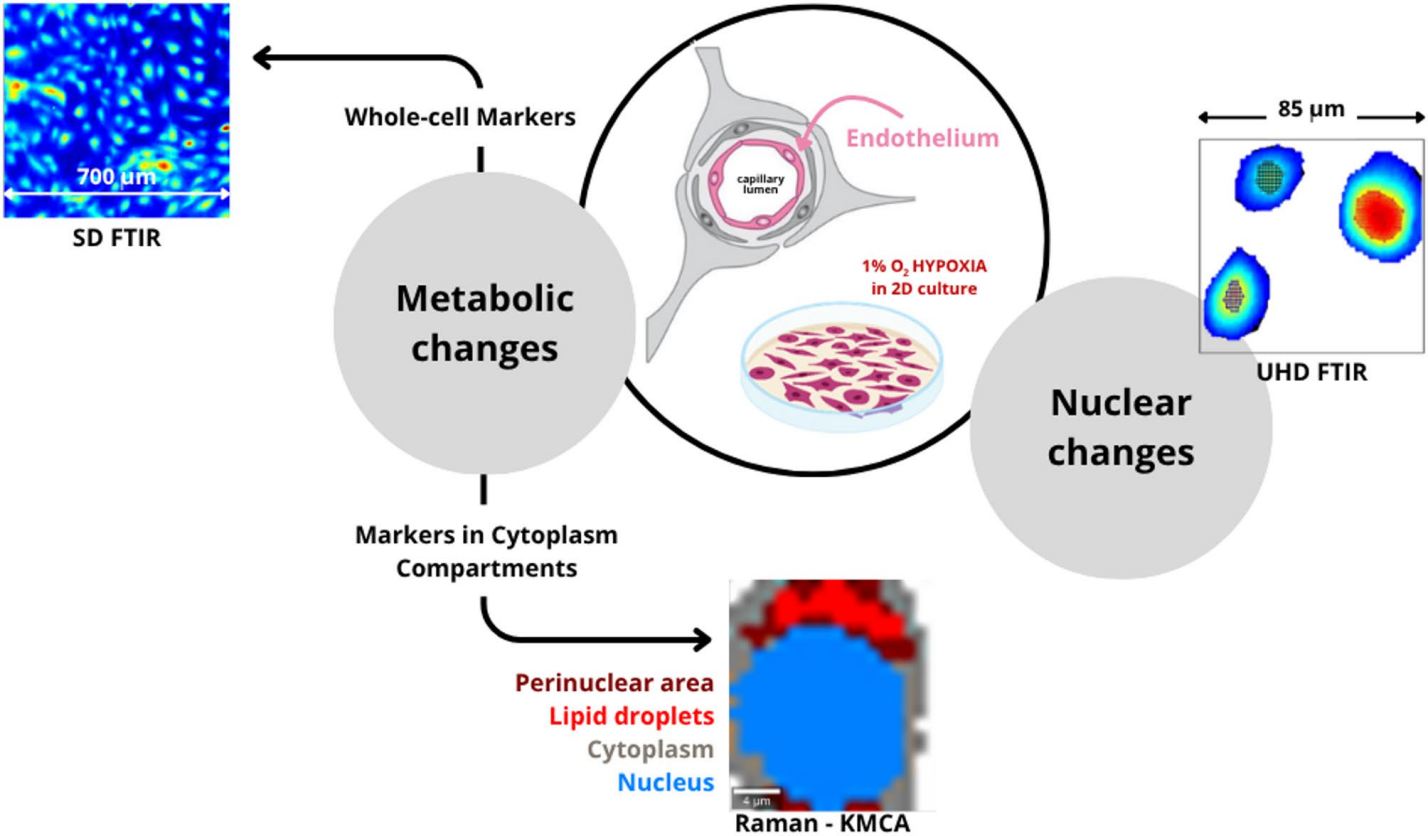
The workflow of FTIR and RS molecular imaging investigations of chronic hypoxia in cerebral microvascular endothelial cells (HBEC 5i). FTIR and RS imaging were utilized to recognize metabolic and nuclear alternations in the whole cell (SD IR imaging—the pixel size of 5.5 μm × 5.5 μm) and its compartments (UHD IR imaging—the pixel size of 0.7 μm × 0.7 μm and RS imaging with a step size of 0.3 μm).

The overall workflow of molecular imaging implemented in this study is presented in Fig. 1. The effect of chronic hypoxia was mimicked by the exposure of cerebral microvascular endothelial cells (HBEC 5i) to 1% O_2_ for 24 h. To avoid intracellular interactions and observe only the single-cell hypoxic response, HBEC 5i cells were cultured in low confluency. Brightfield and fluorescence microscopy showed that the morphology of brain endothelial cells is not affected by the low-O_2_ conditions (Fig. S1 in Supplementary Information). The cells are spindle-shaped and of a similar size. FTIR and RS imaging combined with chemometric methods were utilized to recognize metabolic and nuclear alternations in the whole cell (FTIR) and its compartments (RS).

### Hypoxic effect at the cellular level

**Fig. 2:**
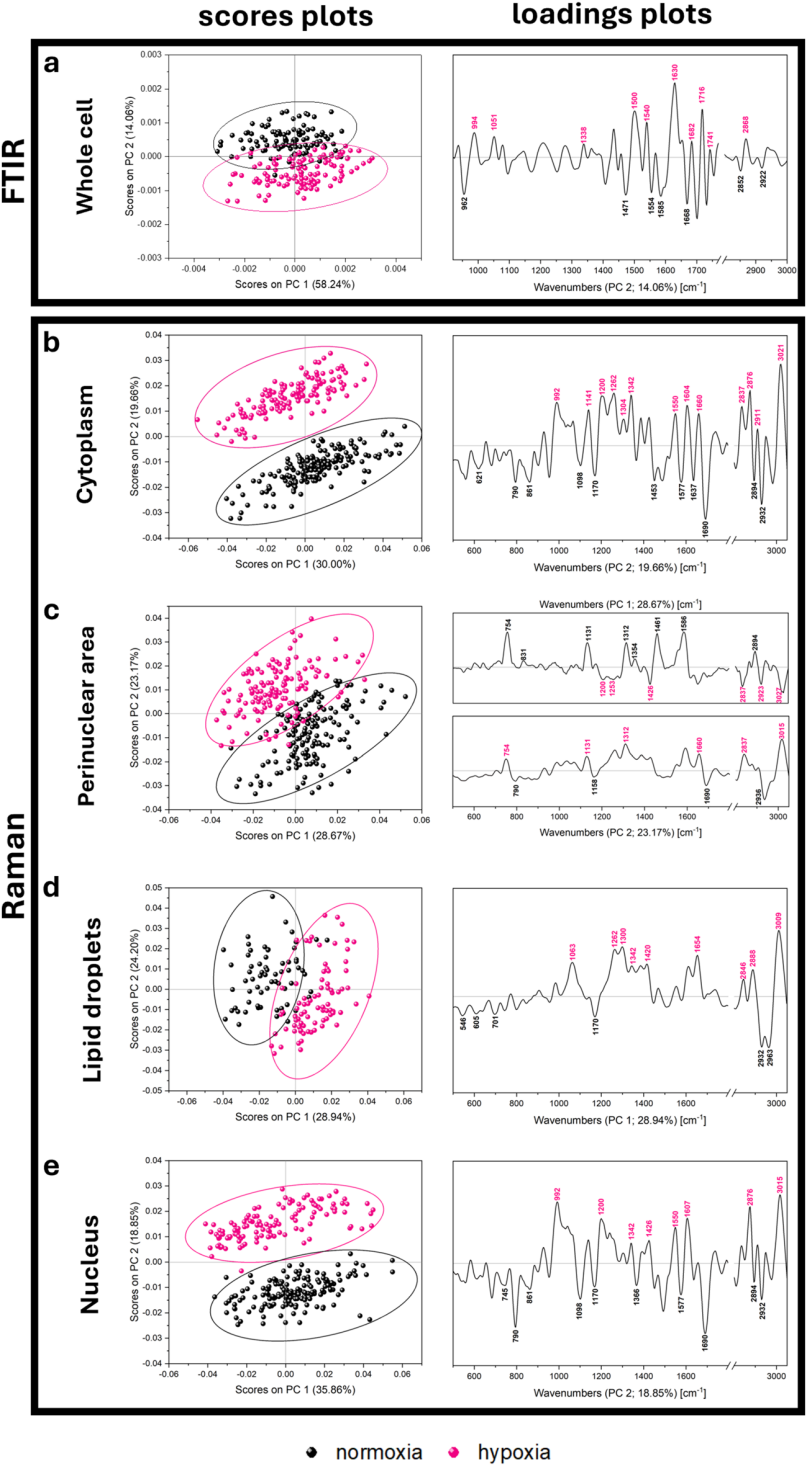
Results of Principal Component Analysis (PCA) performed on (**a**) SD second derivative FTIR spectra of the whole cells and Raman spectra of (**b**) cytoplasm, (**c**) perinuclear area, (**d**) lipid droplets, and (**e**) nuclei. Each point in the scores plots corresponds to one cell.

SD FTIR spectra reveal the presence of the main biocomponents of the investigated cells and represent the overall biochemical information from the whole 120 cells per group (Fig. S2, Tab. S1 in Supplementary Information). Briefly, FTIR spectra demonstrate the presence of nucleic acids (962, 994, 1086, and 1236 cm^−1^), proteins (1300–1682 cm^−1^), and lipids (2852–2960 cm^−1^). Bands from cholesterol (1058 cm^−1^), fatty acids (1392 cm^−1^), and esterified lipids (1165 and 1741 cm^−1^) are also present (Tab. S1 in Supplementary Information). The comparison of the SD FTIR spectra extracted from the single cells reveals that they are sensitive to hypoxia-induced changes in the cellular metabolic network (Fig. S2a in Supplementary Information). These spectra subjected next to Principal Component Analysis (PCA) show the pronounced discrimination between the groups along the PC-2 component with a total variance of 14% (Fig. 2a). A PC-2 loadings plot indicates the main spectral discriminators attributed to nucleic acids (900–1050 cm^−1^), proteins (1500–1700 cm^−1^), and lipids (1700–1750, 2850–2930 cm^−1^) (Fig. 2a and Tab. S1 in Supplementary Information). These results implicate that the rapid and label-free molecular imaging of the whole cells recognizes an impaired synthesis of lipids, changes in DNA:RNA ratio, and the rearrangement of secondary structures of proteins induced by the adaptation of the brain endothelium to low oxygen tension. We discuss them in detail below.

### Markers of hypoxia in the cytoplasm and its structures

K-means clustering of the cytoplasm in the Raman images reveals the presence of a cellular component around the nucleus (further called a perinuclear area, PA) and lipid droplets (LDs) (Fig. 1). Each cellular compartment shows a unique molecular fingerprint displayed in Figs. S2 and S3 in Supplementary Information. The term perinuclear area introduced here mainly includes ER, Golgi apparatus, and mitochondria. This is clustered within the cytoplasm based on the increased intensity of the Raman cytochrome bands (754, 1131, 1312, and 1586 cm^−1^, Figs. S2c and S3 and Tab. S2 in Supplementary Information). PCA scores plots show the clear-cut segregation of both groups at a level of the cytoplasm and its compartments (Fig. 2b–d). The cytoplasm is well separated along PC-2 (19.7% of total variance) (Fig. 2b). The loadings plot indicates the pronounced metabolic changes in the hypoxic cells expressed by the vectors assigned to fatty acids (FAs) (1304, 2837, 2876, 2940 cm^−1^), primarily unsaturated ones (1260, 1660, 3021 cm^−1^), proteins (1141 cm^−1^), and amino acids (1200, 1260, 1342, 1550, 1604 cm^−1^). Over 20% of PC-1 and 2 variance is obtained for the perinuclear area and lipid droplets, indicating that Raman microscopy implicates the induction of specific metabolic changes in these compartments (Fig. 2c, d). The loading vectors of the hypoxic group correspond to the signals of cytochromes and unsaturated FAs, respectively.

**Fig. 3:**
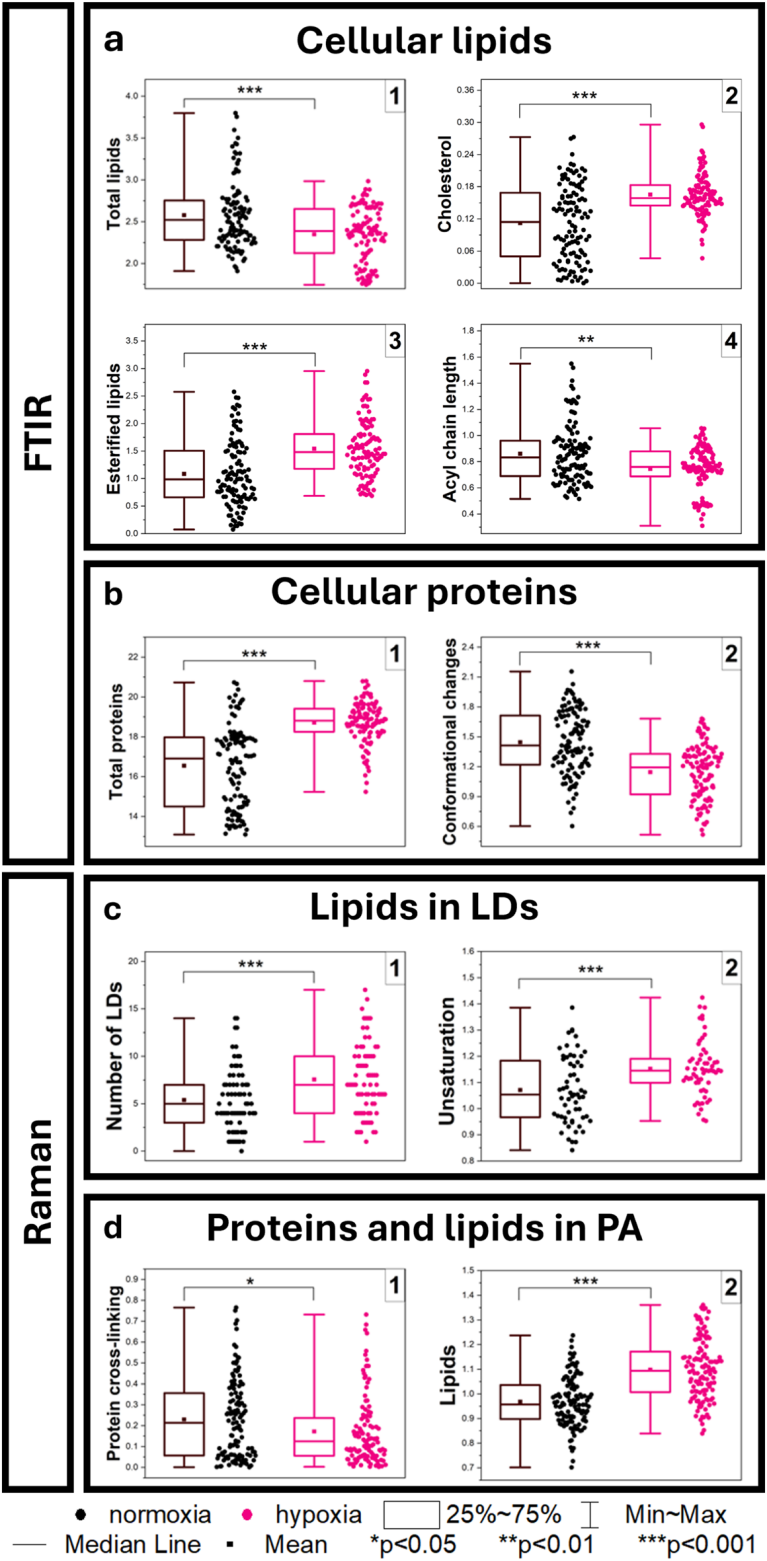
Quantification of the relative amount of (**a**) cellular lipids based on SD second derivative FTIR signals for the total lipids [1], cholesterol [2], esterified lipids [3], and acyl chain length [4]; (**b**) cellular proteins based on SD second derivative FTIR signals for the total proteins [1] and conformational changes [2]; (**c**) lipids in LDs based on Raman signals for the number of lipid droplets [1] and their unsaturation [2]; (**d**) proteins and lipids in PA based on Raman signals for protein cross-linking [1] and lipids [2]. The parameters of the spectral markers are given in Table 1. One-way ANOVA or non-parametric Kruskal–Wallis ANOVA analysis were used to determine to calculated statistical significance at p-value thresholds of *p < 0.05, **p < 0.01, and ***p < 0.001.

We quantified the intensities of the IR and Raman bands to provide spectral ratiometric indicators of the observed processes (Table 1) Under the hypoxic conditions, the HBEC-5i cells reprogram the metabolic processes by the global consumption of lipids (Fig. 3a.1). Other IR lipid-specific signals indicate the synthesis of esterified fatty acids and cholesterol with shorter acyl chains than in normoxia (Fig. 3a.2–4). The primary reservoir of lipids in these cells is lipid droplets and the perinuclear area identified in the Raman images (Fig. 1). The clustering of lipid droplets in the Raman imaging allowed for their quantification per cell (Fig. 3c.1). Although their number in the single cells significantly varies (0 ÷ 18), the median value increases for the hypoxic cells. We also notice that the lipid acyl chains in the LDs become more unsaturated, changing their composition from mono- to polyunsaturated fatty acids according to a calibration curve established by us elsewhere (Fig. 3c.2)^24^. The intensity of choline mode shows a decreasing trend, though without statistical significance (data not shown). Our results imply indeed that cells cultivated in hypoxia produce lipidome alterations comparable to inflammation induced by tumor necrosis factor α (TNF-α) and lipopolysaccharides (LPS) in other endothelial cells like HMEC-1, HMLVEC, and EA.hy926 cells^25–27^. Lipid accumulation under hypoxic conditions is caused by inhibiting fatty acid degradation enzymes leading to lipotoxicity. To avoid this problem, cells convert FAs to neutral esterified lipids accumulated in LDs (Fig. 3a.3, c.1)^28^.

**Table 1:** Summary of the determined spectral markers and their corresponding parameters (band positions in the descriptors and integration ranges in cm^−1^).

	Assignment to biomolecules	Descriptor of the marker	Band integration range
	**Lipids**		
FTIR	Total lipids	(I_2850_ + I_2922_)/I_2960_	I_2850_: 2833–2870; I_2922_: 2903–2945; I_2960_: 2940–2985
	Cholesterol	I_1058_/I_2960_	I_1058_: 1046–1071; I_2960_: as above
	Esterified lipids	I_1740_/I_2960_	I_1740_: 1726–1756; I_2960_: as above
	Phosphorylated lipids	I_1232_/I_2960_	I_1232_: 1213–1258; I_2960_: as above
	Acyl chain length	I_2850_/I_2960_	I_2850_: as above ; I_2960_: as above
	**Proteins**		
	Total proteins	(I_1650_ + I_1540_)/I_2960_	I_1650_: 1612–1668; I_1540_: 1524–1565; I_2960_: as above
	Conformational changes	I_1650_/I_1540_	I_1650_: as above; I_1540_: as above
	Serine (Ser) and threonine (Thr)	I_1172_/I_2960_	I_1172_: 1132–1190; I_2960_: as above
	Tyrosine (Tyr)	I_1513_/I_2960_	I_1513_: 1480–1525; I_2960_: as above
	**Nucleic acids**		
	DNA	I_962_/I_2960_	I_962_: 947–977; I_2960_: as above
	RNA	I_994_/I_2960_	I_994_: 981–1014; I_2960_: as above
	DNA/RNA	I_962_/I_994_	I_962_: as above; I_994_: as above
Raman	**Lipids**		
	Unsaturation of lipid droplets	I_1660_/I_1440_	I_1660_: 1608–1714; I_1440_: 1400–1505
	Lipids	I_2850_/I_1440_	I_2850_: 2820–2863; I_1440_: as above
	**Proteins**		
	Protein cross-linking	I_1040_/I_1005_	I_1040_: 1025–1065; I_1005_: 986–1030
	**Nucleic acids**		
	DNA	I_790_/I_1440_	I_790_: 770–810; I_1440_: as above

The next lipid and cytochrome-rich compartment is the perinuclear area. First of all, the PC-1 and 2 scores plots indicate the substantial contribution of the cytochrome Raman signals (754, 1131, 1312, and 1586 cm^−1^) to the discrimination of both groups (Fig. 2c). However, the intensity of these bands does not indicate a statistically significant difference (data not shown). The additional k-means clustering of the perinuclear region revealed the presence of granules with a high accumulation of cytochromes, and their number decreased by 6% under hypoxic conditions (Tab. S3 in Supplementary Information). So, we conclude that the mitochondrial activity of the brain endothelium was suppressed due to oxygen depletion. Interestingly, the decreased ratio of the aromatic amino acid residues also shows the loss or alternation of the stability of protein hydrophobic interior and transmembrane segments in this region of the cells accompanied by the accumulation of lipids (Fig. 3d.1 and 2)^29^. These observations are congruent with disturbances in mitochondrial functions and ER stress in chronic hypoxia^30^. Hypoxia limits the supply of oxygen available to accept electrons in fatty acid oxidation, resulting in mitochondrial injury and the formation of LDs in the damaged mitochondria and endoplasmic reticulum^31,32^.

Apart from the lipid composition, we notice considerable differences in the protein-specific region of the FTIR spectrum of the whole cells (Fig. S2a in Supplementary Information). First of all, the quantification of the amide I and II bands indicates the synthesis of proteins upon the hypoxic stress (Fig. 3b.1). Furthermore, the hypoxic condition modifies the secondary structures of proteins as the ratio of Amide I: Amide II band intensity shows (Fig. 3b.2). This is congruent with the appearance of the sub-bands at 1630 and 1682 cm^−1^ around the major amide I at 1649 cm^−1^ attributed the dominant α-helical secondary structure (Fig. S2a in Supplementary Information). The new amide I band exhibits the formation of β-sheets (1630 cm^−1^) and intramolecular aggregation of proteins (1682 cm^−1^). It has been suggested that the reduction in these Raman signals may reflect conformational changes in ER proteins and/or qualitative or quantitative alterations in molecules involved in the ER stress response^32^.

### Markers of hypoxia in the nucleus

**Fig. 4:**
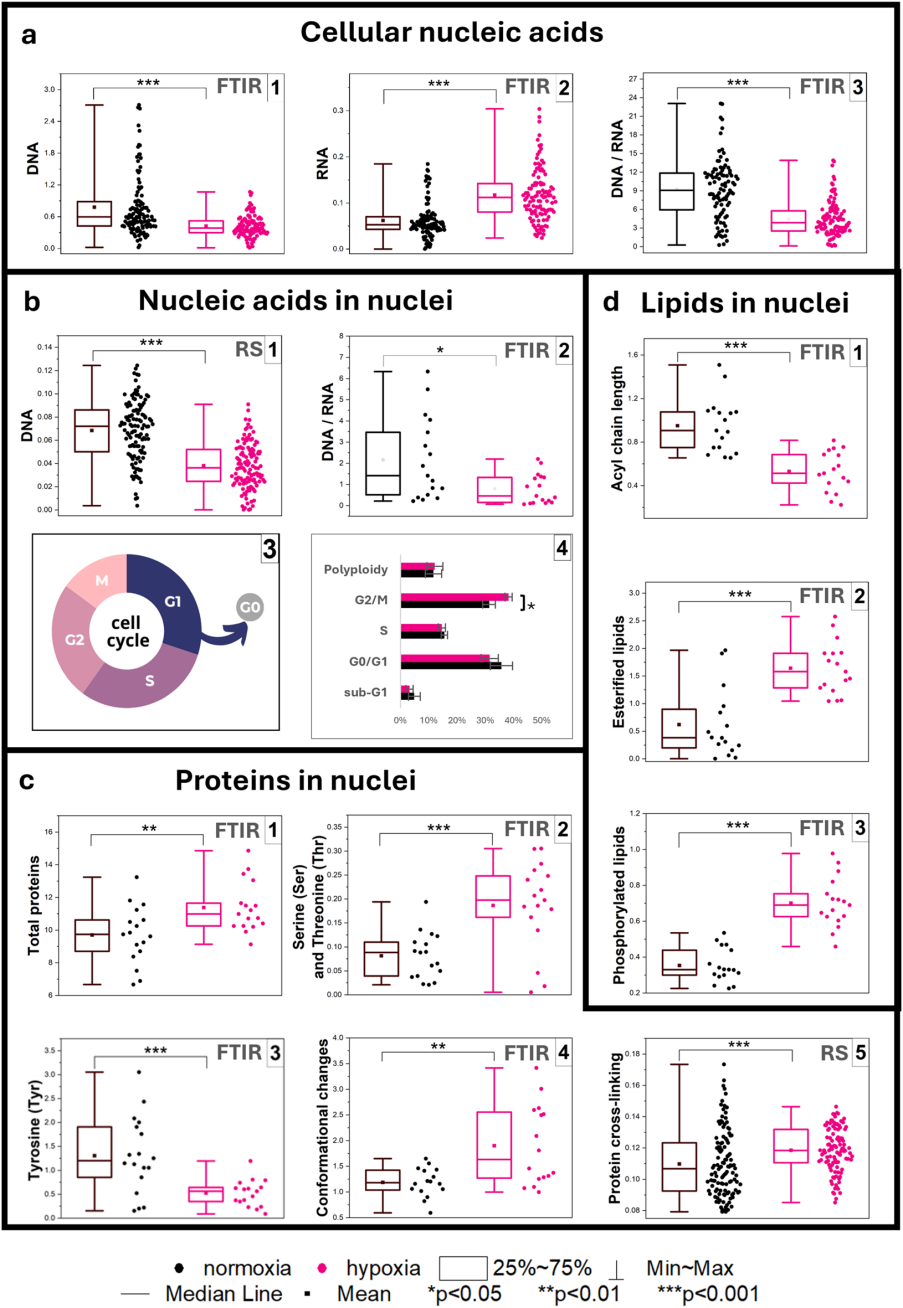
Quantification of the relative amount of (**a**) cellular nucleic acids based on SD second derivative FTIR signals for the DNA [1], RNA [2] and the DNA/RNA ratio [1]; (**b**) nucleic acids in nuclei based on Raman signals DNA [1] and based on UHD second derivate FTIR signals for the DNA/RNA ratio [2], phases of the cell cycle [3], and percentage of cell cycle phases + /− SEM (SEM, Standard Error of the Mean) [4]; (**c**) proteins in nuclei based on UHD second derivative FTIR signals for total proteins [1], serine (Ser) and threonine (Thr) [2], tyrosine (Tyr) [3], conformational changes [4] and based on Raman signals for protein cross-linking [5]; (**d**) the total lipids in nuclei based on UHD second derivative FTIR signals for acyl chain length [1], esterified lipids [2], and phosphorylated lipids [3]. The parameters of the spectral markers are given in Table 1. One-way ANOVA or non-parametric Kruskal–Wallis ANOVA analysis were used to determine to calculated statistical significance at p-value thresholds of *p < 0.05, **p < 0.01, and ***p < 0.001.

FTIR spectra gathered in SD IR imaging of whole cells also show significant changes in nucleic acids. The quantification of nucleic acid markers indicates a decrease in the DNA level, increase in RNA synthesis and concomitant decrease of the DNA:RNA ratio (Fig. 4a.1–3, Table 1).

The use of UHD IR imaging with an FPA pixel size of 0.67 μm × 0.67 μm and Raman cluster of the nucleus confirms that these changes exclusively appear in the nucleus (Figs. S4b.1–2 and S5 in Supplementary Information). The decrease in the DNA content is indeed associated with the degradation and condensation of chromatin. This observation is congruent with hypoxia-induced DNA fragmentation into oligonucleosomal fragments (internucleosomal cleavage) reported by Ferrer and Zubrow^33,34^. Other IR bands of DNA in the nuclei of the stressed cells are down-shifted by 2–3 cm^−1^, i.e. 1057 [ν(C–O)_deoxyribose_] and 1234 cm^−1^ [ν_as_(PO_2_^−^)] (Fig. S4a in Supplementary Information). Such shifts have been observed in the B → A transition of DNA due to dehydration or damage to its secondary structures^35^.

The increased absorbance of the RNA band, in turn, indicates the upregulation of transcription processes that leads to the increased production of proteins (Figs. 4c.1 and S5.2 in Supplementary Information, Table 1). Interestingly, nuclear proteins also undergo compositional transformations highlighted by an increased content of proteins with Ser and Thr residues but a lower level of Tyr-containing proteins (Fig. 4c.2–3). We attributed these markers to serine/threonine kinases, cell cycle, and HIF-1α regulators, whose overexpression has been observed in the hypoxic environment^36,37^. In addition, the ratios of amide I/II bands in the FTIR spectra and Raman bands of aromatic AAs indicate pronounced modifications of structures of the protein scaffolds in the chromatin (Fig. 4c.4–5).

The stress induced by hypoxia and reduced oxygen availability affects the cell cycle, a crucial process through which cells replicate their genetic material^38^. It has been proposed that FTIR spectroscopy can be used to monitor the cell cycle, as changes in the DNA/RNA and protein regions indicate specific cell cycle phases^39,40^. The analysis of the cell cycle reveals that in hypoxia, the percentage of cells in the G2/M phase was higher than that in normoxia (Fig. 4b.3–4). During the G2 phase, the synthesis of proteins essential for mitosis occurs (Fig. 4c.1). The observed increase in RNA synthesis, resulting from the production of mRNA and other RNA molecules required for mitosis, leads to a concurrent decrease in the DNA:RNA ratio (Fig. S5.2 in Supplementary Information and Fig. 4b.2)^41^.

The FTIR spectra of the nuclei also show an up-shift and broadening of the 2850 and 2920 cm^−1^ bands attributed to the stretching vibrations of the methylene groups in the acyl chains of lipids; mainly glycerophospholipids in the nuclear membrane (Fig. S4 in Supplementary Information)^42^. This observation indicates the conformational disordering process of the fatty acid acyl chains and is accompanied by their significant shortening (Fig. 4d.1)^43^. Nuclear lipids form microdomains whose composition is adjusted to cellular processes associated with newly synthesized RNA as it is observed here in the endothelium exposed to oxygen depletion^42^. Moreover, the membrane and matrix fluidity act as platforms for the regulation of gene expression, including DNA duplications well as fragmentation^44,45^. In addition, the synthesis of esterified and phosphorylated lipids is up-regulated in hypoxia and results from their established role in the modulation of the protein activity and ion channels in the membrane^46^ (Fig. 4d.2–3).

Interestingly, PC-2 for the Raman spectra of the nuclei shows pronounced segregation of both groups (a total variance of ca. 20%), c.f. (Fig. 2e). Positive vectors attributed to the cells exposed to oxygen depletion indicate the significant alternations of signals of pyrimidine and purine bases (T—745 cm^−1^, C—992 cm^−1^, T/C—1426 cm^−1^, G—1550 cm^−1^, C/G—1607 cm^−1^), (polynucleotide chain—1342 cm^−1^), amino acid residues (Hyp/Tyr—1200 cm^−1^), and lipids (2876, 3015 cm^−1^). The appearance of the discriminators assigned to cytosine and guanine has been reported as a predictor of DNA methylation in abnormal cells^47,48^. Since DNA methylation is associated with genetic modifications, this PCA result supports the observation of such massive metabolic and genetic changes detected here for the hypoxic effect on the brain endothelium.

**Fig. 5:**
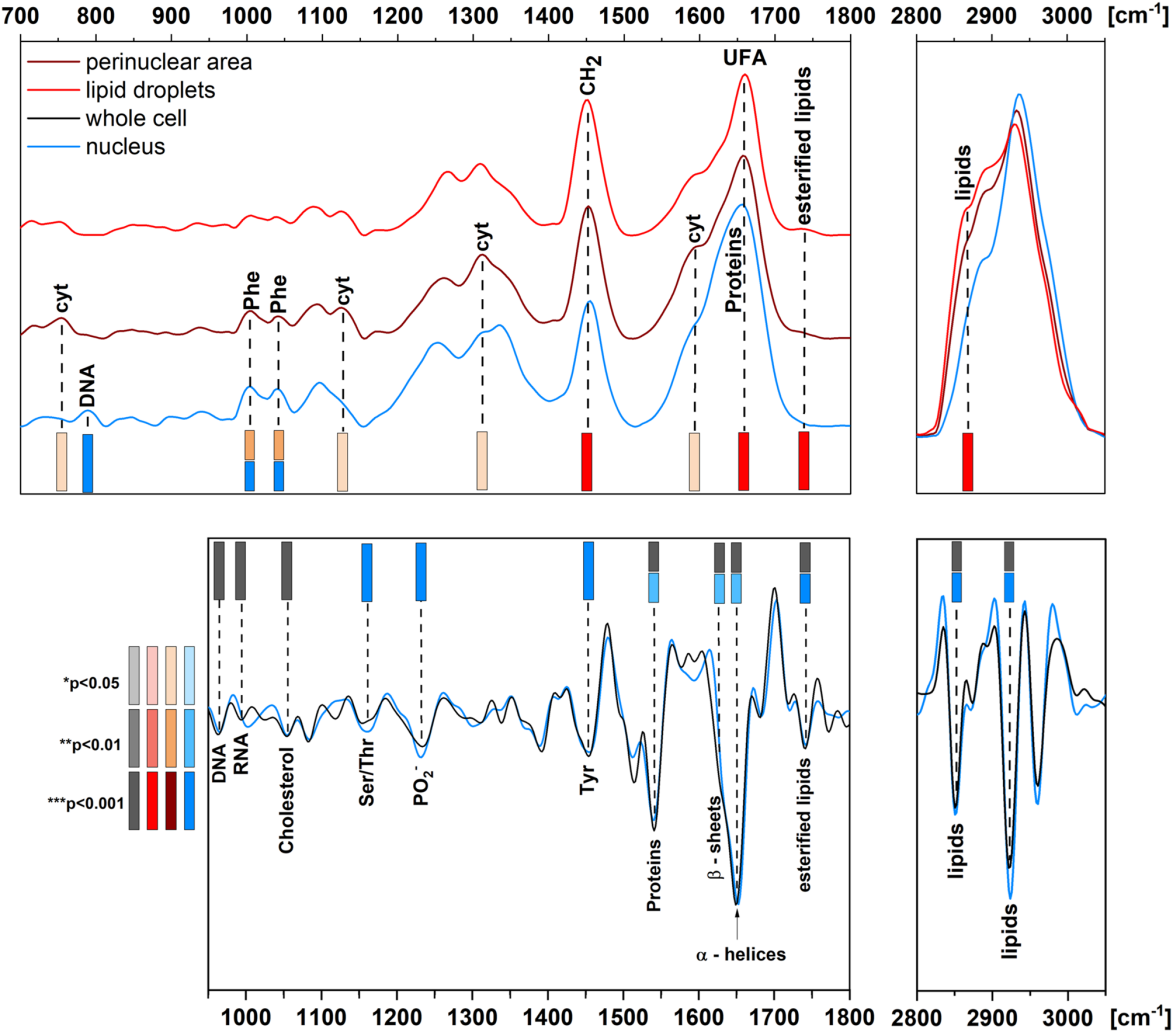
A Raman (upper) and second derivative IR (down) based barcode of hypoxia in cerebral microvascular endothelial cells. The discriminant wavenumbers are represented by different color gradients using *p*-values: max *p* > 0.001, min *p* < 0.05.

To summarize our findings, we illustrated the determined spectral markers as a barcode for easy-to-interpret valuable information (Fig. 5). The statistical analysis of spectral discriminators provided a gradient of IR and Raman barcoding. This code also showed that data can be collected in specific spectral regions, shortening acquisition time and reducing spectral data. In the case of the FTIR spectra, the highly discriminant features appeared in the regions of 950–1000, 1500–1700, and 2800–2970 cm^−1^. Considering the need for rapid monitoring of the induced hypoxic effects in cells, one can only employ the registration of FTIR spectra from the cell centers, which are often attributed to the nucleus since the SD IR imaging mode showed a similar spectral pattern as UHD. Although Raman-based barcoding showed relevant stripes at wavenumbers at 750, 790, 1005, 1040, 1131, 1312, 1440, 1586, 1660, 1740, and 2850 cm^−1^ assigned to biomolecules like hemoproteins, aromatic amino acid residues, and the olefin group, their unique assignments to biochemical processes in specific compartments complete the IR-based description of the hypoxic effect. The Raman mapping of numerous cells is time-consuming; however, discrete-frequency imaging techniques like stimulated and coherent anti-Stokes Raman microscopies provide significant chemical contrast localizing lipids droplets, mitochondria, proteins, and nucleic acids.

## Conclusions

Prolonged oxygen depletion induces damage in cerebrovascular microcirculation and is primarily determined by the expression of the HIF family in in vitro experiments. This study demonstrates that FTIR and RS spectroscopic imaging recognizes several biochemical events appearing in the cellular compartments of the HBEC 5i cells exposed to 1% O_2_ concentration for 24 h. They can be exploited by unsupervised principal component analysis and signal intensities in the defined wavenumber regions as a barcode of the hypoxic response. The PCA level of differentiation of the whole cells and their compartments is high (14–30%). Simultaneously, the intensity changes are significant (mostly *p* < 0.001) and implicate content and structural modifications of proteins and lipids, degradation of DNA, lipotoxicity, and reduced mitochondrial activity. To evaluate, in total, the spectral signature of the hypoxic stress, both IR and Raman spectroscopy are needed. Raman microscopy resolves the intracellular compartments and identifies the saturation degree of lipids, phenylalanine, and cytochromes in the cells in their natural environment. In turn, IR microscopy gives a sensitive response to structural changes in secondary structures of proteins, nucleic acids, and lipids.

The continuous technological advances in Raman and FTIR microscopy, particularly those utilizing single laser wavelengths for rapid probing of live cells, hold great potential for real-time hypoxia monitoring. Our results give a barcode of these frequencies that could be useful for temporal and spatial detection of this dysfunctional status in the brain endothelium. This code may be dependent on the type of endothelial cell lines and needs to be verified. Standardization of protocols for cell preparation and measurement protocols is also critical for obtaining similar hypoxia fingerprinting results. Furthermore, the chemical and physical inhomogeneity of some cells can generate interference effects, such as scattering and fluorescence, confounding the established markers. The data pre-processing and analysis steps proposed here constitute a solid workflow for further applications of the IR/RS metrics, but their robustness should be validated through inter-laboratory ring trials.

Finally, one must consider that molecular changes occurring during hypoxia are complex, transient, and often occur at multiple levels simultaneously. So, using a single methodology can give an incomplete or misleading picture. Integrative approaches that combine multiple methodologies are undoubtedly necessary to fully understand the cellular response to hypoxia. Apart from HIV immunostaining, other targeted proteins or the morphology of organelles can be detected using fluorescence microscopy. At the same time, bioassays, such as qPCR, glycomics, or lipidomics, despite their destructive nature, can provide insight into the roles of nucleic acids, carbohydrates, and lipids.

## Supplementary Information

Supplementary Information.

## Data Availability

All data supporting the findings of this study are available within the paper and its Supplementary Information and have been deposited in RODBUK—Cracow Open Research Data Repository (accessible at 10.57903/UJ/JLRZ8Y). Supplementary data related to this article are available online.
