# Supplementary material for: Marker-independent vibrational spectroscopy imaging recognizes the hypoxia effect in the human brain endothelium
Source: Sci Rep. 2025 Jul 18;15:26112. doi: 10.1038/s41598-025-11000-2 (PMC12274419; doi:10.1038/s41598-025-11000-2)
Supplement: Supplementary file 1 — Supplementary Information. [file 41598_2025_11000_MOESM1_ESM.pdf]

Supplementary Information

Marker-independent vibrational spectroscopy imaging recognizes the hypoxia effect in the human brain endothelium

Aleksandra Pragnąca[1,2+], Anna Antolak[1+], Zuzanna J. Krysiak[3,4], Monika Leśniak[3], Agata Borkowska [3], Robert Zdanowski[3], Kamilla Malek[1]

[1] Jagiellonian University in Krakow, Faculty of Chemistry, Department of Chemical Physics, Gronostajowa 2, 30-387, Krakow, Poland

[2] Jagiellonian University in Krakow, Doctoral School of Exact and Natural Sciences, prof. S. Lojasiewicza 11 Street, 30-348 Krakow, Poland

[3] Military Institute of Medicine National Research Institute, Laboratory of Molecular Oncology and Innovative Therapies, Szaserow 128 Street, 04-141 Warsaw, Poland

[4] Department of Biosystems and Soft Matter, Institute of Fundamental Technological Research, Polish Academy of Sciences, Pawińskiego 5B, Warsaw, 02-106, Poland

[*]corresponding author: kamilla.malek@uj.edu.pl

[+]these authors contributed equally to this work

Keywords: hypoxia, brain endothelium, FTIR and Raman spectroscopy imaging,

ORCID of the authors

Aleksandra Pragnąca: 0000-0003-3156-4194

Anna Antolak: 0000-0002-8008-8684

Zuzanna Krysiak: 0000-0002-9913-5479

Monika Leśniak: 0000-0003-0340-4054

Agata Borkowska: 0000-0002-3273-4610

Robert Zdanowski: 0000-0003-0455-1072

Kamilla Malek: 0000-0003-0582-2743

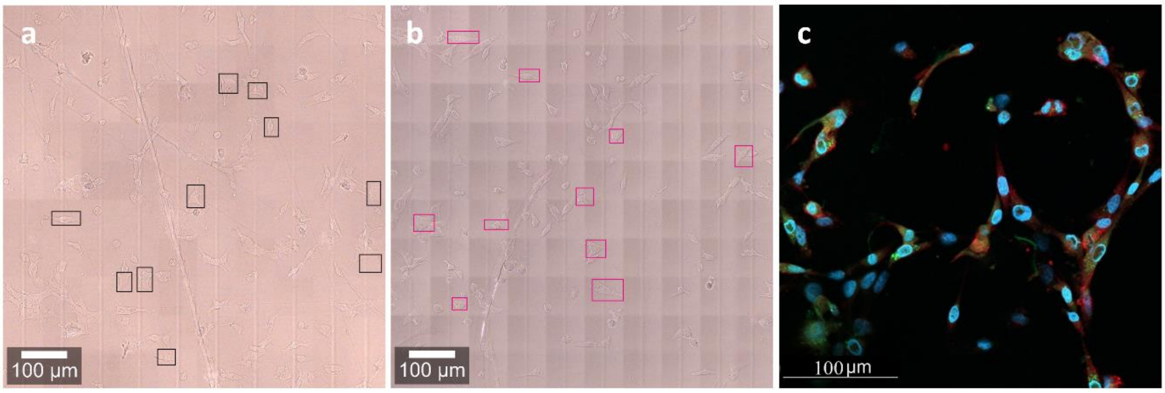

Fig. S1 Exemplary images of HBEC-5i brain endothelial cells: (a) bright-field image of normoxic cells; (b) bright-field image of hypoxic cells; (c) fluorescence images (20x) of normoxic cells stained with Hoechst 33342 (nuclei - blue), BODIPY (lipids - green), and Phalloidin-Atto 488 (F-actin - red) dyes.

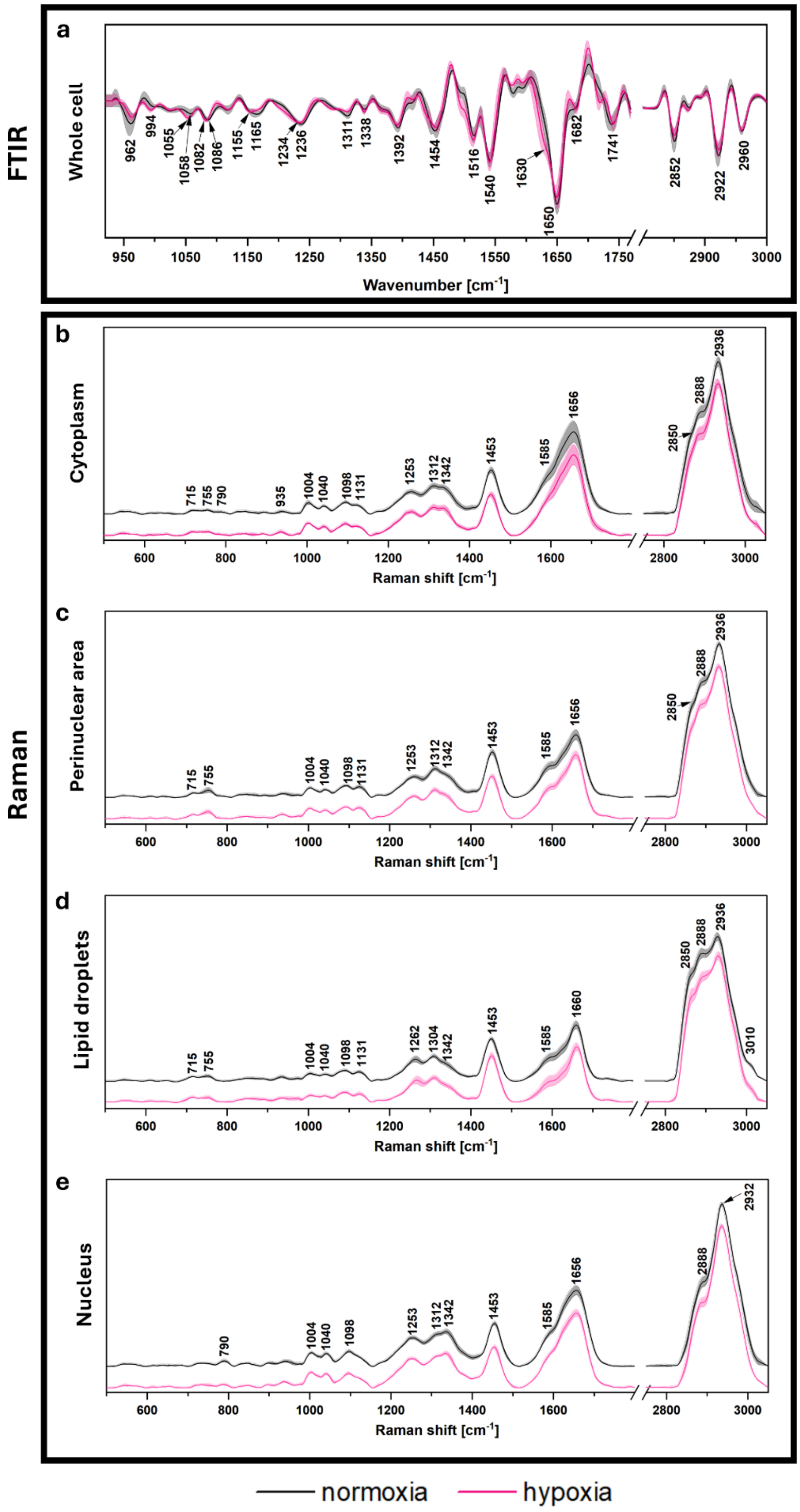

Fig. S2 Average spectra of HBEC 5i under normoxic (black) and hypoxic (pink) conditions calculated from (a) SD second derivative FTIR spectra of the whole cells (920-1750, 2800-3000 cm^{-1}) and Raman spectra (500-1800, 2800-3050 cm^{-1}) of (b) cytoplasm, (c) perinuclear area, (d) lipid droplets, and (e) nuclei. Shading represents ±SD.

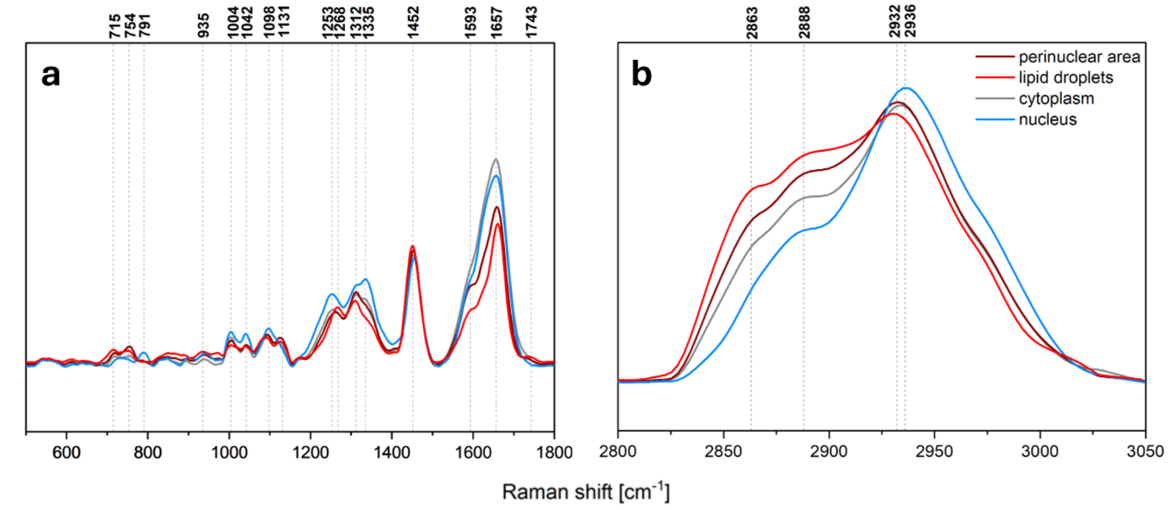

Fig. S3 Mean Raman spectra of the cellular compartments extracted from false-color k-means (KMC) map calculated from the Raman images of the single cells (see Fig. 1) in the regions of 500-1800 (a), and 2800-3050 cm^{-1} (b). The marked band positions are specific for a given compartment; their assignments are summarised in Table S1. Color code: nucleus – blue, cytoplasm - grey, perinuclear area - brown, lipid droplets – red.

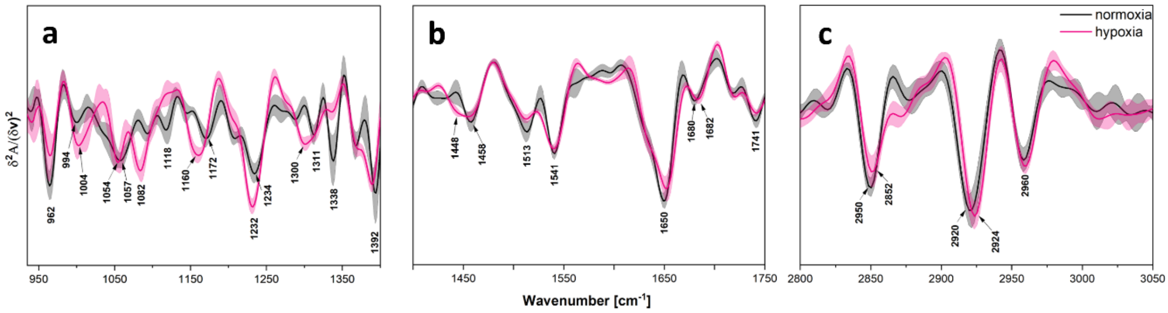

Fig. S4 Average second derivative FTIR spectra (UHD mode) of the nuclei of the cells in the regions of 920-1400 (a), 1400-1750 (b), and 2800-3000 cm^{-1} (c). Shading represents ±SD.

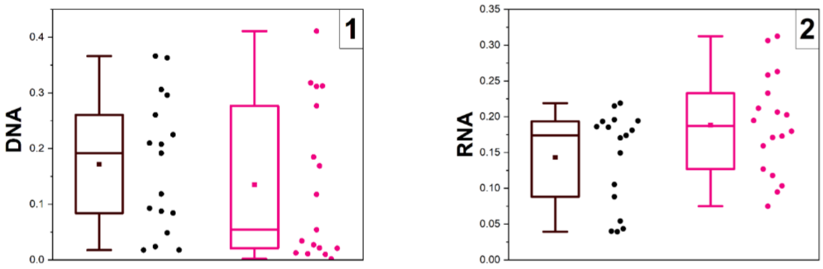

Fig. S5 Quantification of nucleic acids in nuclei based on UHD second derivative FTIR signals for the DNA [1: (I_{964}/I_{2960})], and RNA [2: I_{994}/I_{2960}].

Table S1. FTIR band positions and their assignments to vibrational modes of biomolecules [1–6].

Band position [cm^{-1}]	*Assignment to biomolecules and vibrational modes*
**962**	DNA; backbone ν(C-C)
**994-1004**	RNA
**1051**	DNA; backbone ν(C-O)
**1054-1057**	DNA; $\nu(C\text{-}O)_{deoxyribose}$
**1058**	Cholesterol
**1082-1086**	Nucleic acids; $\nu_s(PO_2^-)$<br>Phospholipids; $\nu_s(PO_2^-)$
**1118**	Ribose (RNA); ν(C-O)
**1155-1165**	Esterified lipids; ν_{as}(CO-O-C)
**1172**	Ser and Thr (proteins)
**1232-1236**	Nucleic acids; $\nu_s(PO_2^-)$<br>Phospholipids; $\nu_s(PO_2^-)$
**1300**	Proteins (amide III); δ(N-H), ν(C-N)
**1311**	Phospholipids; $\nu(CH_2)$
**1338**	Lipids, proteins; $\delta(CH_2)$
**1392**	Free fatty acids, amino acids; $\nu_s(COO^-)$
**1448-1458**	Proteins; $\delta(CH_2, CH_3)$
**1471**	Lipids, proteins; $\delta(CH_2)$
**1500**	Proteins (amide II); δ(N-H) and ν(C-N)
**1513-1516**	Tyr (proteins); ν(CC) of the ring
**1540**	Proteins (amide II); δ(N-H) and ν(C-N)
**1554**	Proteins (amide II); δ(N-H) and ν(C-N)
**1585**	Lipids, proteins, nucleic acids; $\nu_{as}(COO^-)$, ν(C=N), ν(C=C)
**1630**	β-sheets in proteins (amide I); ν(C=O) and δ(N-H)
**1650**	α-helices in proteins (amide I); ν(C=O) and δ(N-H)
**1668**	3_{10}-helices in proteins (amide I); ν(C=O) and δ(N-H)
**1680-1682**	intramolecular aggregation of proteins (amide I); ν(C=O) and δ(N-H)
**1716**	Fatty acids; $\nu_{acid}(C=O)$
**1741**	Esterified lipids; $\nu_{ester}(C=O)$
**2852**	Long chain of fatty acids; $\nu_s(CH_2)$
**2868**	Lipids, proteins; $\nu_s(CH_3)$
**2920-2924**	Lipids, proteins; $\nu_{as}(CH_2)$
**2960**	Lipids, proteins; $\nu_{as}(CH_3)$

ν – stretching mode, as – asymmetric, s – symmetric; δ – in-plane deformations; Ser – Serine; Thr – Threonine; Tyr – tyrosine

Table S2. Raman band positions and their assignments to vibrational modes of biomolecules [1,6–9].

Band position [cm^{-1}]	*Assignment to biomolecules and vibrational modes*
**546**	Cholesteryl esters; $\delta(CH_2)$ in ring
**605**	Esterified lipids; δ(C-O-C)
**621**	Proteins (Phe); γ(C-C)
**701**	Cholesterol, cholesteryl esters; cholesterol ring def.
**715**	Phospholipids; ν(C-N)
**745**	T (nucleic acids); ring breathing, δ(C-H)
**754**	Cyt. c and c_1; ν(porphyrin ring)
**790**	Nucleic acids; $\nu_s(PO_2)$
**831**	Hyp/Tyr (amino acids);

Band position [cm^{-1}]	*Assignment to biomolecules and vibrational modes*
**861**	Pro, Hyp, Tyr (proteins); ν(C=C)
**992**	C (nucleic acids)
**1005**	Phe (proteins); ring breathing
**1040**	Cross-linked Phe (proteins)
**1063**	Lipids, proteins; ν(C-C)
**1098**	Phosphate-containing molecules; $\nu_s(PO_2)$
**1131**	Phospholipids; ν(C-C)<br>*Cyt. c and c_1; ν(porphyrin ring)
**1141-1158**	Proteins; $\nu(CH_3/CH)$
**1170**	Tyr (proteins); δ(C-H)
**1200**	Hyp/Tyr (amino acids)
**1253**	Proteins (amide III); ν(C-N), δ(N-H), $\nu(CH_3\text{-}C)$
**1262**	Unsaturated lipids; δ(=CH)
**1300**	Fatty acids and triacylglycerols; $\tau(CH_2/CH_3)$
**1304**	Fatty acids and triacylglycerols; $\tau(CH_2/CH_3)$
**1312**	G (nucleic acids); ring breathing, δ(C-H)<br>*Cyt. c and c_1; δ(C-H)
**1342**	Nucleic acids, proteins; ring breathing; δ(C-H)<br>Lipids; $\tau/\delta(CH_2/CH_3)$
**1354**	G (nucleic acids)
**1366**	Lipids; $\delta(CH_2)$
**1420**	Lipids; $\delta(CH_2)$
**1426**	T/C (nucleic acids)
**1440**	Lipids; $\delta(CH_2, CH_3)$
**1453**	Proteins, lipids; $\delta(CH_2, CH_3)$
**1461**	Cholesteryl esters; $\delta(CH_2, CH_3)$
**1550**	Trp, G; ν(C=C)
**1577**	G (nucleic acids); ring breathing modes
**1586**	A, G (nucleic acids); ring breathing<br>*Reduced cyt. c, c_1 and b; ν(methine bridges – C_aC_m,C_aC_mH bonds)
**1604**	A/G (nucleic acids)
**1607**	C/G (nucleic acids); $\nu(NH_2)$
**1637**	Proteins (amide I), α-helix and β-structure
**1654-1660**	Fatty acids, proteins; ν(C=C)
**1690**	Proteins (amide I); ν(C=O)
**1740**	Esterified lipids; ν(C=O)
**2837**	Lipids; $\nu_s(CH_2)$
**2846-2850**	Lipids, fatty acids; $\nu_s(CH_2)$
**2876-2888**	Lipids, proteins; $\nu_{as}(CH_2)$
**2894**	Lipids; $\nu_s(CH_3)$
**2911**	Lipids, proteins; $\nu(CH_3)$
**2923**	Lipids; $\nu_s(=CH_3)$
**2932-2940**	Lipids, proteins; $\nu_{as}(CH_3)$
**2963**	Nucleic acids, lipids; $\nu_{as}(CH_3)$, $\nu_{as}CH(-CH_2)$
**3009-3027**	Lipids, fatty acids; ν(=CH)

ν – stretching mode, as – asymmetric, s – symmetric; δ – in-plane deformations; γ - out-of-plane deformations; τ – twisting; cyt – cytochromes; A – adenine; C – cytosine; G – guanine; T – thymine; Phe – phenylalanine; Tyr – tyrosine; Trp - tryptophan; Hyp – hydroxyproline; *Bands at 1131, 1312, and 1586 cm^{-1} are assigned to cytochromes only if the ~750 cm^{-1} band is present.

Table S3. The number of subcellular compartments and whole cells detected by Raman and FTIR imaging of endothelial cells under normoxia and hypoxia conditions.

Raman Imaging				
Classes from KMCA	*normoxia (N=120)*	*% normoxia*	*hypoxia (N=120)*	*% hypoxia*
**Cytoplasm**	115	96%	116	97%
**Perinuclear area**	98	82%	112	93%
**Cytochromes**	67	56%	60	50%
**Lipid droplets**	49	41%	74	62%
**Cell nucleus**	87	81%	103	86%
FTIR Imaging				
**Whole cells**	120		120	

References

1. Augustyniak, K. *et al.* Adipose-derived mesenchymal stem cells' adipogenesis chemistry analyzed by FTIR and Raman metrics. *J. Lipid Res.* **65,** (2024).
2. Augustyniak, K. *et al.* Molecular tracking of interactions between progenitor and endothelial cells via Raman and FTIR spectroscopy imaging: a proof of concept of a new analytical strategy for in vitro research. *Cell. Mol. Life Sci.* **80,** 329 (2023).
3. Kujdowicz, M. *et al.* Ftir spectroscopic imaging supports urine cytology for classification of low-and high-grade bladder carcinoma. *Cancers (Basel).* **13,** (2021).
4. Banyay, M., Sarkar, M. & Gräslund, A. A library of IR bands of nucleic acids in solution. *Biophysical Chemistry* **104,** 477–488 (2003).
5. Whelan, D. R. *et al.* Monitoring the reversible B to A-like transition of DNA in eukaryotic cells using Fourier transform infrared spectroscopy. *Nucleic Acids Res.* **39,** 5439–5448 (2011).
6. Molony, C. *et al.* Label-free discrimination analysis of de-differentiated vascular smooth muscle cells, mesenchymal stem cells and their vascular and osteogenic progeny using vibrational spectroscopy. *Biochim. Biophys. Acta - Mol. Cell Res.* **1865,** 343–353 (2018).
7. Czamara, K. *et al.* Raman spectroscopy of lipids: A review. *Journal of Raman Spectroscopy* **46,** 4–20 (2015).
8. Talari, A. C. S., Movasaghi, Z., Rehman, S. & Rehman, I. U. Raman spectroscopy of biological tissues. *Applied Spectroscopy Reviews* **50,** 46–111 (2015).
9. Roman, M., Wrobel, T. P., Panek, A., Paluszkiewicz, C. & Kwiatek, W. M. Lipid droplets in prostate cancer cells and effect of irradiation studied by Raman microspectroscopy. *Biochim. Biophys. Acta - Mol. Cell Biol. Lipids* **1865,** (2020).
